# Long term Follow-up of Transvaginal Anatomical Implant of Mesh in Pelvic organ prolapse

**DOI:** 10.1038/s41598-018-21090-w

**Published:** 2018-02-12

**Authors:** De-Yi Luo, Tong-Xin Yang, Hong Shen

**Affiliations:** 1 Department of Urology, West China Hospital, Sichuan University, Chengdu, China; 2 Department of Urology, Institute of Urology, West China Hospital, Sichuan University, Chengdu, China; 3 Department of Urology, The Second Affiliated Hospital of Kunming Medical University, Kunming, China

## Abstract

Transvaginal mesh (TVM) is a minimally invasive but effective treatment for pelvic organ prolapse (POP). However, mesh exposure is a common and problematic complication after TVM. This study assessed the safety and long-term outcomes of TVM. A retrospective review was performed on the medical records of 175 consecutive patients who underwent TVM with the anatomical implant technique for pelvic organ prolapse at our center from April 2007 to December 2012. All operations were performed using TVM with the anatomical implant technique. Intraoperative variables, postoperative complications, and TVM outcomes were assessed. In average of 8 years (ranging from 4 to 10 years), the objective cure ratio reached 99.4%; and the subjective success rate of the TVM operation was 91.4%. Only 2 cases (1.1%) were identified as having mesh exposure. The reoperation rate was 4.0% (95% CI, 1.1–6.9%). No patients abstained from sex due to the operation or postoperative discomfort. Our anatomical implant technique for correcting POP is feasible in TVM procedures, which lead to favourable subjective and objective outcomes with the lowest rates of mesh exposure (1.1%) in published data. Therefore, performing TVM operations with the appropriate technique could consider to be permitted.

## Introduction

Transvaginal mesh (TVM) is a minimally invasive but effective treatment for pelvic organ prolapse (POP); and it has become a simple and widely used surgical technique^1,2^. The introduction of polypropylene mesh, however, was accompanied by several specific surgical complications: mesh exposure, mesh-associated infections and mesh-stimulated tissue reactions, etc.^3,4^. Therefore, the U.S. Food and Drug Administration (FDA) issued warnings against applying TVM in 2008 and 2011, then issued orders to manufacturers of urogynecologic surgical mesh devices to conduct postmarket surveillance studies to address specific safety and efficacy concerns related to TVM after 2012. In December 2015, SCENIHR (Scientific Committee on Emerging and Newly Identified Health Risks) recommended that TVM should only be used when other surgical procedures had previously failed or were expected to fail due to increased risks associated with the use of synthetic mesh (mesh exposure rates range from 4% to 19%)^5^.

In an era that promotes minimally invasive surgery to decrease morbidity and hospitalisation costs, TVM remains an attractive option. To improve the efficacy and minimize the surgical complications of TVM, we proposed a modified vaginal dissection technique (anatomical implant technique) for pelvic reconstruction. This study evaluated the feasibility of the anatomical implant technique and reported the long-term outcomes. The data from the study should be considered when choosing an appropriate POP therapy, especially among medical decision support systems.

## Methods

This study was a retrospective cohort analysis, and after receiving approval from the local ethics committee (West China Hospital, Sichuan University), informed consent was obtained from all patients. All methods in this study were performed in accordance with the relevant guidelines and regulations.

### Preoperative preparation and instrumentation

A consecutive series of 175 patients who underwent TVM for POP at our center between April 2007 and December 2012 were included. Patient demographics and clinical characteristics were reported in Table 1. Operations were performed on adult females, with completed child-bearing and in post-menopausal stage, who also had stage II to IV POP based on POP-quantification (POP-Q). Patient POP grades were reported in Table 2, followed by the ICS and IUGA^6^. The available TVM surgical kits were Gynecare Prolift® and Gynecare Prosima® (Ethicon, Sommerville, NJ, USA). The mesh was monofilament, type I and macroporous. All operations were performed by the experienced surgeon using the ANATOMICAL IMPLANT TECHNIQUE; and the standard method of mesh insertion was based on the product’s design^7,8^. Local vaginal oestrogen preparation was not mandatory.

**Table 1:** Pre- and post-operative subjective symptoms.

n	total	p**
	175	
Concomitant continence procedure	29	
Age (y)	64.5 ± 11.4	
BMI (kg/m2)	23.1 ± 2.5	
Parturition (time)	4.1 ± 1.8	
II stage	56(32.0)	
Pre-operative POP-Q staging (ICS)a III stage	94(53.7)	
IV stage	25(14.3)	
Pelvic prolapse symptomb (pre-/post-)	102(58.3)/8(4.6)	<0.001
Postoperative chronic pain	17(9.7)	
Storage symptoms (pre-/post-)	87(49.8)/34(19.4)	<0.001
Occult urinary incontinencec	22(15.1)	
Voiding symptoms (pre-/post-)	87(49.8)/2(1.1)	<0.001
Constipation or diarrhoea (pre-/post-)	19(10.9)/6(3.4)	0.007

^a^The highest part of prolapse staging, ^b^n (%), similarly hereinafter, ^c^based on patients who did not have concomitant continence procedure.

**Comparisons of patient reported outcomes between pre- and post-operation.

**Table 2:** POP-Q in Patients, n (%).

POP-Q /staging (n = 175)	Pre-operative					Post-operative				
	0	I	II	III	IV	0	I	II	III	IV
Aa	6(3.4)	8(4.6)	110(62.9)	51(29.1)	0	79(45.1)	87(49.7)	8(4.6)	1(0.6)^a^	0
Ba	3(1.7)	0	63(36.0)	87(49.7)	22(12.6)	79(45.1)	87(49.7)	8(4.6)	1(0.6)	0
Ap	6(3.4)	43(24.6)	84(48.0)	44(24.0)	0	88(50.3)	84(48.0)	3(1.7)	0	0
Bp	5(2.9)	39(22.3)	78(44.6)	36(20.6)	17(9.7)	88(50.3)	84(48.0)	3(1.7)	0	0
C	1(0.6)	33(18.9)	68(38.9)	49(28.0)	24(13.7)	151(86.3)	24(13.7)	0	0	0
D	1(0.7)	47(31.3)	63(42.0)	26(17.3)	13(8.7)	143(100)	0	0	0	0

^a^The recurrent prolapse of this patient restored to stage I after a ASC further operation.

### Patient positioning

A urethral catheter was inserted under general anaesthesia. Patients were placed in a dorsal lithotomy position, with the legs were supported by Allen stirrups and the arms were tucked beside the body, ensuring that pressure points were padded. Patients were then appropriately prepped and set in an 80° Trendelenburg position.

### Anatomical implant technique

**Figure 1:**
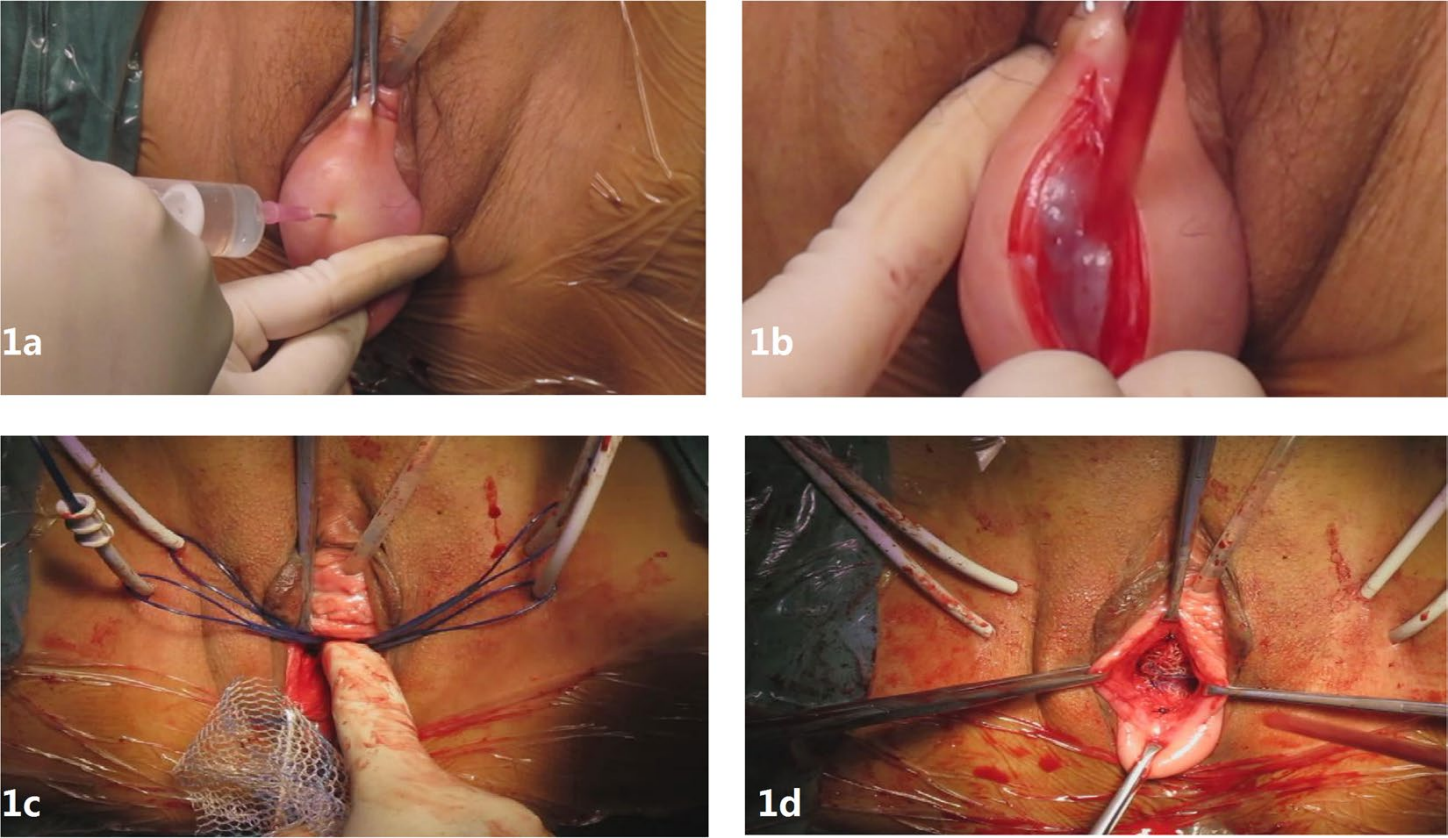
Surgical treatment of POP: (**a**) water injection into pelvic fascia, (**b**) dissection of full-thickness vaginal wall, (**c**) cannulas in place, (**d**) anterior implant in place.

**Figure 2:**
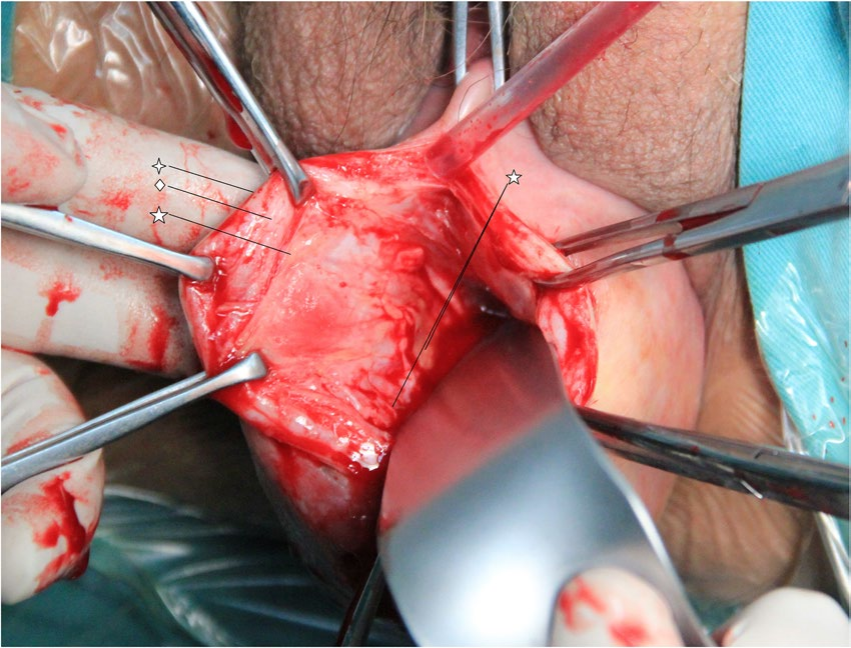
Lamina propria, muscularis and adventitia of vaginal wall.  Lamina propria of the vaginal wall consisting of non-keratinized stratified squamous epithelium. ◇ Smooth muscle of the vaginal wall. ☆ The adventitia is a variably discrete layer of collagen, elastin, and adipose tissue containing blood vessels, lymphatic vessels, and nerves. The capillary network within the vaginal adventitia is crucial for tissue activity and healing the vaginal wall.

Well-performed hydrodissection beneath vaginal adventitia is crucial to preserve the capillary network within the vaginal adventitia and for placing the mesh into the correct layer. During TVM procedures, the mesh was placed under the adventitial layer (beneath the full thickness of the vaginal wall), in the monolayer and continuous space consisting of visceral endopelvic fascia and vesicovaginal or rectovaginal adventitia. In details, 30–50 ml of normal saline was injected forward, left and right through one needle inserted to the middle line of the vaginal wall (Fig. 1a). The hydrodissection target layer was the potential septum beneath the vaginal adventitia. The vaginal wall was characterized by wide range swelling with normal surface after injection into the right layer, indicating that a water sac formed beneath the full thickness of the vaginal wall and the blood supply was appropriate (Fig. 2). If the injection failed to reach the correct layer, then only a focal water vacuole would be formed in the lamina propria or muscularis; and this area of the vaginal wall would become thin and white.

After hydrodissection, a vertical incision was made at vaginal wall to the water sac (Fig. 1b). Haemorrhaging could be seen until the incision penetrated the vaginal adventitia into the water sac layer; then clear, normal saline would leak from the incision without bleeding. Next, the water sac was cut to the left and right beside the vaginal adventitia to make a space for the mesh, clearing the operation field so the bleeding could be controlled. Notably, the capillary network can be seen in the transparent, thin layer of fascia on the back of the vaginal adventitia after a well-performed hydrodissection.

Four skin incisions were made on the genitocrural crease: two in the anteromedial edge of the obturator foramen at the level of the urethra and the other two at 2 cm below and 1 cm lateral to the first ones. Bilateral passage of the two upper cannula-equipped guides at 1–2 cm of the prepubic portion of the arcus tendinous fascia pelvis (ATFP) and bilateral passage of the two lower cannula-equipped guides at 1–2 cm of the distal portion of the ATFP (1 cm from the ischial spine) allowed each prosthetic arm to be passed through the obturator foramen (Fig. 1c). Next, the mesh was positioned tension-free under the bladder (Fig. 1d) and was fixed to the bladder neck and uterine isthmus with a Prolene suture to support the uterus. Posterior implant placement was performed similarly to the anterior implant per the manufacturer’s instructions.

Electrocauterization was not performed during the operation, as it would damage the capillary network. Clipping the haemorrhagic area using tissue forceps is appropriate when incising the vaginal wall. A well-performed hydrodissection ensures a clear procedural field. A standardized procedure minimizes the surgical time,and adequately controls blood loss. The estimated blood loss was calculated by the suction volume in post-operation plus the estimated Laps blood loss (1 lap rinse equal to 10 ml).

### Postoperative follow-up

Follow-ups were completed in all the patients from December, 2016 to June, 2017. Objective success was defined as the lowest point of prolapse never reaching the level of the hymen (point 0). Subjective success was defined when the patient reported the absence of a bulge and the absence of unacceptable symptoms, such as pain or incontinence^6^. The Chinese versions of the Pelvic Floor Distress Inventory (PFDI-20)^9–11^and the Patient Global Impression of Improvement (PGI-I)^12^ were used to quantify the outcomes, while the Visual Analogue Scale (VAS)^13^ was used to quantify pain, if any.

### Data analysis

Statistical analysis was performed using the Statistical Package for the Social Sciences (SPSS) 19.0 for Windows (IBM, New York, NY). Unless specified, normally distributed continuous parameters were presented as the mean ± SD (standard deviation) and analysed using paired Student’s t-test in the pre- and postoperative comparison; non-normal distribution parameters were presented as median (range); Categorical parameters were presented as number (percentage) and analysed using chi-square test. A p-value of less than 0.05 was considered statistically significant.

## Results

Among the 175 patients, the mean age was 64.5 ± 11.4, the average parturition was 4.1 ± 1.8, the median duration of disease was 3 years (range from 1 month to 50 years, and a quartile of 1 year and 10 years) and the median follow-up was 8 years (range from 4 to 10 years). 12 patients (6.9%) had a surgical history of total or subtotal hysterectomy due to benign causes. Through In correlation analysis, patient age distribution was positively correlated with each point of POP-Q (p < 0.001), but duration of disease, BMI and parturition times were not (p > 0.10). In addition, pelvic prolapse symptoms were positively correlated with each POP-Q point (p < 0.001).

The anatomical implant technique was applied in all patient operations. 36 cases of Prolift A, 114 cases of Prolift T, 4 cases of Prolift P, 3 cases of Prosima A and 18 cases of Prosima C were performed; 25 cases of Tension-free Vaginal Tape Obturator (TVT-O) and 4 cases of Tension-free Vaginal Tape (TVT) were performed at the same period of surgery. The operation duration was 62.1 ± 17.1 minutes, and estimated intra-operative blood loss was 64.5 ± 46.1 ml. The reoperation rate was 4.0% (95% CI, 1.1–6.9%). Two bladder perforation cases were discovered during the operation. No intra-operative complications occurred in the remaining patients.

The postoperative POP-Q point distribution was improved (p < 0.001) from the pre-operation (Table 2). At a median of 8-year follow-up, only 1 patient had a relapsed prolapse (after Prosima C), and the objective cure ratio reached 99.4%.

The average postoperative hospital stays were 4.0 ± 1.0 days, and urethral catheters were usually removed 2 days after the operation. Two cases (1.1%) were verified to have mesh exposure on the vaginal wall.

After TVM, patients’ subjective symptoms were improved (Table 1), and the subjective success rate of the TVM operation performed in 175 patients was 91.4% (95% CI, 87.3–95.6%). 17 patients reported chronic pain and discomfort on the perineum, operative incision or puncture area (9.7%; 95% CI, 5.3–14.1%) (Table 3). 24 patients with an average age of 50.0 ± 7.2 years were sexually active, in which 22 patients reported an improved quality of postoperative sex, and no patients abstained from sex due to postoperative discomfort (Table 3).

**Table 3:** Peri- and post-operative negative events or description (n = 175).

Events or descriptions	n	Code	Re-intervention	Follow-up^a^
Intra-operative bladder perforation	2	4 C/T1/S3	One ureter reimplantation was performed 3 days after the initial surgery.	Normal renal function and normal cystoscopy results.
Left common peroneal nerve injury	1	7B/T1/S3	Conservative treatment and rehabilitation for 50 days.	The sensory and movement function of left lower limb recovered to normal.
Pelvic hematoma	1	7 A/T2/S3	Conservative treatment.	Pelvic hematoma cured.
Transvaginal incision delayed healing	2	1 Cd/T2/S1 1Bd/T2/S1	Conservative treatment for one case, re-sutured for another in office.	Transvaginal incision healed well.
POP recurrence	1	—	ASC was performed 9 months after the initial surgery.	Further surgery success.
Vaginal wall mesh exposure	2	3Aa/T4/S1 2 C/T3/S1	Expectant and follow-up.	One case with small amount of vaginal discharge.
Bladder wall mesh exposure	1	4 A/T4/S3	Holmium laser cystoscopy lithotripsy and remove of foreign body	Symptoms relieved after further operation.
Frequency, urgency and nocturia	10	4B/T3/S1	Expectant and follow-up, behaviour intervention or oral medication.	No severe frequency occurrence with limited impact on quality of life.
Urinary incontinence	26	4B/T3/S1	Further surgery for severe incontinence (4 cases during follow-up) and expectant or conservative treatment for others.	Symptoms controlled by re-interventions.
Unusual vaginal discharge	4	1 C/T3/S1 1 Cc/T3/S1	Expectant and follow-up without mesh exposure.	Limited impact on quality of life.
Urinary tract infection	3	4B/T3/S1	Oral antibiotics.	Cured.
Dysuria	2	4B/T3/S1	Expectant and follow-up.	Limited impact on quality of life.
Long-term constipation or diarrhoea	6	—	Expectant and follow-up or behaviour intervention.	Limited impact on quality of life.
Pelvic organ straining feeling	5	—	Expectant and follow-up.	Symptoms could be relieved after rest and no prolapse recurrence with limited impact on quality of life.
Abdomen, perineal or transvaginal incision chronic pain or discomfort (VAS scored of 1 or above)	17	1B/T3/S1 6B/T3/S4	Expectant or conservative treatment.	6 cases VAS scored of 2 or above and reported serious impact on their quality of life.
Dyspareunia	2	1Bc/T3/S1	Expectant and follow-up.	Limited impact on quality of life.

^a^The impact on quality of life was determined by patient reported outcomes, which is the definition of subjective success. Accordingly, 15 cases reported unsuccessful outcomes.

**Figure 3:**
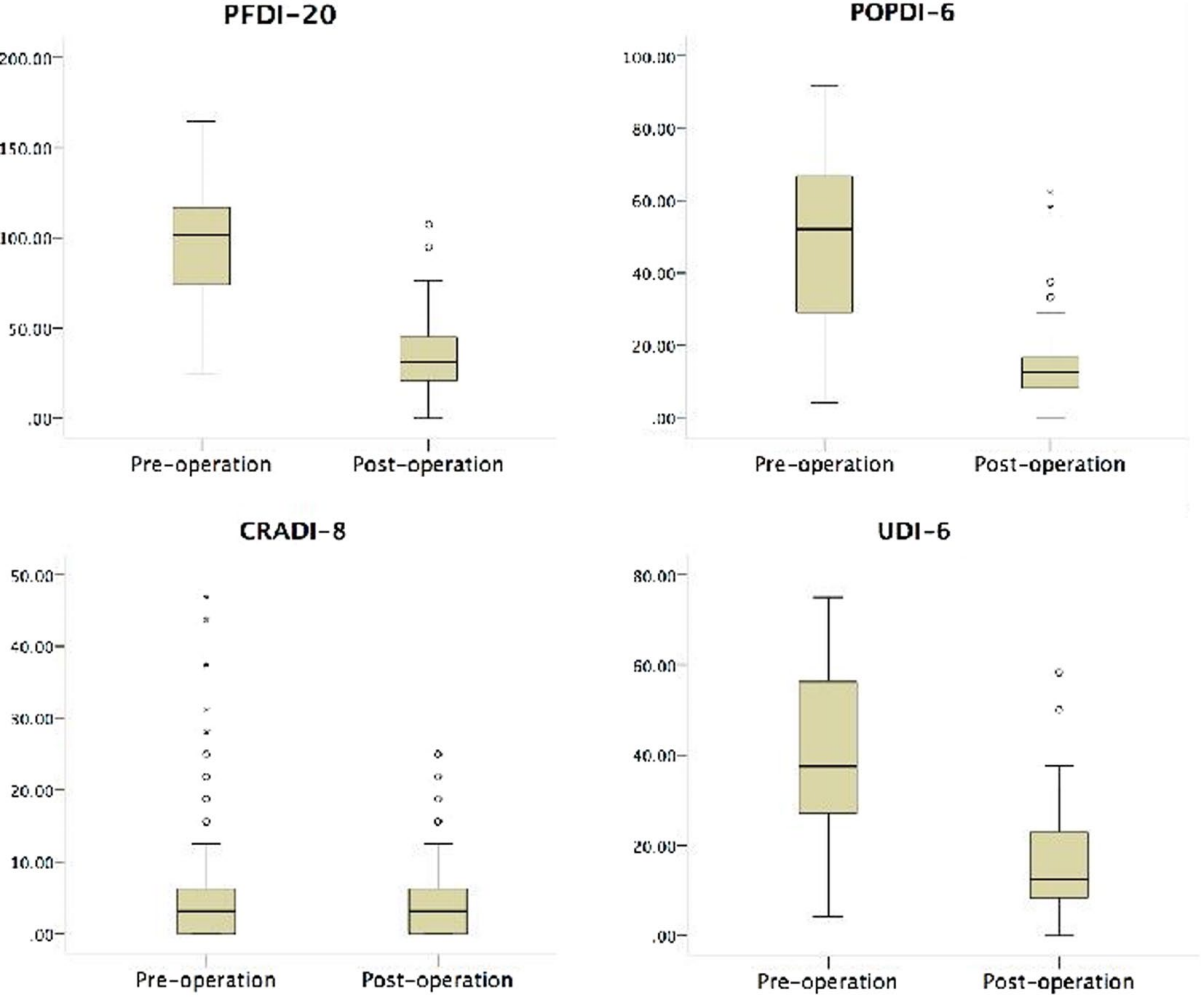
Chinese version of the Pelvic Floor Distress Inventory (PFDI-20) was used to quantify the outcomes. The PFDI-20 consists of the Pelvic Organ Prolapse Distress Inventory (POPDI-6), the Colorectal-Anal Distress Inventory (CRADI-8) and the Urinary Distress Inventory (UDI-6) with a perfect score of 300.

Ninety-six PFDI-20 questionnaires (54.9%) were effectively completed with a total of 97.0 ± 32.1 and 34.6 ± 19.0 points for pre-operation and post-operation, respectively (p < 0.01) (Fig. 3). The average postoperative PGI-I score was 1.43 ± 0.71 with statistical improvement (95% CI: 1.32–1.54) in comparison with preoperative PGI-I. When the patients were asked whether they would have the operation again with the clear knowledge of their postoperative life state, the responses were all positive; and they would recommend this operation to other patients.

## Discussion

This study was conducted at a single high-volume center to describe the anatomical implant technique. The operation’s objective success rate was 99.4%, and the subjective success rate was 91.4%. Only 2 cases (1.1%) were verified to have mesh exposure without symptoms. Our results demonstrate that the transvaginal anatomical implant technique is effective and safe. Therefore, performing the TVM operation using the appropriate technique should be considered to permit.

The unique advantages of this vaginal surgery include, having no incision in the abdominal wall; being performed via the abdominal cavity; having a short operating time and a fast postoperative recovery; and being relatively safe for older and chronic patients. Synthetic mesh application enhances the success rate of the vaginal pelvic reconstructive operation^14–17^, solving the postoperative problems of the traditional repair operation, such as diminished vaginal volume and dysuria, and achieves the goal of recovering pelvic functions^14,16,18^. However, long-term retention of the synthetic mesh may result in complications^19^. Therefore, some experts believe that the TVM operation should not be recommended after weighing the advantages and disadvantages; because although it may result in a better recovery, but the related complications generate other problems^3,4^.

A primary complication of the synthetic mesh is mesh exposure. Some research shows that the mesh exposure rate is between 4% and 19%^14,20^. Only 2 patients (2/175, 1.1%) experienced mesh exposure in our study, which is the lowest in the published data on TVM. This mesh exposure rate is even lower than that of ACS (3%)^21^ or LSC (2.5%)^21^.

The anatomical implant technique aims to maintain the integrity of all vaginal wall layers; and their blood is supplied by placing the mesh in the correct anatomical space. The full thickness of the vaginal wall is composed of three layers: the lamina propria, muscularis, and adventitia. The adventitia is a variable, discrete layer of collagen, elastin, and adipose tissue containing blood vessels, lymphatic vessels, and nerves. The capillary network within the vaginal adventitia is crucial for the tissue activity and healing ability of the vaginal wall. Stiffness and mesh exposure in the vaginal wall are related to the damaged capillary network within the vaginal adventitia. Therefore, the TVM procedure should minimize injury to this area, which helps to decrease haemorrhaging and to preserve the intact blood supply to the vaginal wall, ensuring tissue vitality and preventing possible complications from the mesh.

In surgical terms, the layer exposed after dissecting between the lamina propria and muscularis in the anterior vaginal wall is the “pubocervical fascia” ^22,23^, and the same layer in the posterior vaginal wall is the “rectovaginal fascia” ^24,25^. Traditional prolapse repair surgery for POP is performed based on these fascias. TVM procedures place the mesh under the adventitial layer (beneath the full thickness of the vaginal wall), in the monolayer and continuous space consisting of the visceral endopelvic fascia and the vesicovaginal or rectovaginal adventitia, but different from the fascias in traditional prolapse repair. Otherwise, a portion of the mesh will remain under the lamina propria layer or in the muscularis of the vaginal wall, rendering the vaginal wall incomplete.

Mesh exposure is also related to the surgeon’s experience^25^. We devote ourselves to standardizing and improving our operational skills, and we believe that positive technical exchange and training reduces the incidence of mesh exposure. Through follow-up visits with 175 patients, the lower mesh exposure rates should be attributed to proper operating skills. We cannot conclude that the main factor in reducing mesh exposure is the anatomical implant technique; however, this technique has better postoperative outcomes, and thus requires further study. We also expect that more clinical research will report the TVM experiences of the patients.

## Conclusions

TVM procedures are feasible with favourable prospects for POP. The anatomical implant technique has low mesh exposure rates that are close to zero.

## References

[1] Maher CM, Feiner B, Baessler K, Glazener CM (2011). Surgical management of pelvic organ prolapse in women: the updated summary version Cochrane review. Int Urogynecol J..

[2] Feiner B, Jelovsek JE, Maher C (2009). Efficacy and safety of transvaginal mesh kits in the treatment of prolapse of the vaginal apex: a systematic review. BJOG..

[3] Withagen MI, Milani AL, den Boon J (2011). Trocar-guided mesh compared with conventional vaginal repair in recurrent prolapse: a randomized controlled trial. Obstet Gynecol..

[4] Sivaslioglu AA, Unlubilgin E, Dolen I (2008). A randomized comparison of polypropylene mesh surgery with site-specific surgery in the treatment of cystocoele. Int Urogynecol J Pelvic Floor Dysfunct..

[5] SCENIHR (Scientific Committee on Emerging and Newly Identified Health Risks), The safety of surgical meshes used in urogynecological surgery, 3 December 2015.

[6] Toozs-Hobson P (2012). An International Urogynecological Association (IUGA)/International Continence Society (ICS) joint report on the terminology for reporting outcomes of surgical procedures for pelvic organ prolapse. Int Urogynecol J..

[7] Lucioni A (2008). The surgical technique and early postoperative complications of the Gynecare Prolift pelvic floor repair system. Can J Urol..

[8] Sayer T (2012). Medium-term clinical outcomes following surgical repair for vaginal prolapse with tension-free mesh and vaginal support device. Int Urogynecol J..

[9] Barber MD, Walters MD, Bump RC (2005). Short forms of two condition-specific quality-of-life questionnaires for women with pelvic floor disorders (PFDI-20 and PFIQ-7). Am J Obstet Gynecol..

[10] Chan SS (2013). Responsiveness of the Pelvic Floor Distress Inventory and Pelvic Floor Impact Questionnaire in women undergoing treatment for pelvic floor disorders. Int Urogynecol J..

[11] Chan SS (2011). Chinese validation of Pelvic Floor Distress Inventory and Pelvic Floor Impact Questionnaire. Int Urogynecol J..

[12] Srikrishna S, Robinson D, Cardozo L (2010). Validation of the Patient Global Impression of Improvement (PGI-I) for urogenital prolapse. Int Urogynecol J..

[13] Ulrich D, Guzman Rojas R, Dietz HP, Mann K, Trutnovsky G (2014). Use of a visual analog scale for evaluation of bother from pelvic organ prolapse. Ultrasound Obstet Gynecol..

[14] de Landsheere L (2014). Changes in elastin density in different locations of the vaginal wall in women with pelvic organ prolapse. Int Urogynecol J..

[15] Nguyen JN, Burchette RJ (2008). Outcome after anterior vaginal prolapse repair: a randomized controlled trial. Obstet Gynecol..

[16] Vollebregt A, Fischer K, Gietelink D, van der Vaart CH (2011). Primary surgical repair of anterior vaginal prolapse: a randomised trial comparing anatomical and functional outcome between anterior colporrhaphy and trocar-guided transobturator anterior mesh. BJOG..

[17] Maher C, Feiner B, Baessler K, Schmid C (2013). Surgical management of pelvic organ prolapse in women. Cochrane Database Syst Rev..

[18] Nieminen K (2010). Outcomes after anterior vaginal wall repair with mesh: a randomized, controlled trial with a 3 year follow-up. Am J Obstet Gynecol..

[19] Jacquetin B, Cosson M (2009). Complications of vaginal mesh: our experience. Int Urogynecol J Pelvic Floor Dysfunct..

[20] de Tayrac R, Sentilhes L (2013). Complications of pelvic organ prolapse surgery and methods of prevention. Int Urogynecol J..

[21] Nygaard IE (2004). Abdominal sacrocolpopexy: a comprehensive review. Obstet Gynecol..

[22] Weber, A. M., Walters, M. D., Piedmonte, M. R., Ballard LA. Anterior colporrhaphy: a randomized trial of three surgical techniques. Am J Obstet Gynecol. **185****:**1299-304; discussion 304-6 (2001). 10.1067/mob.2001.119081 11744900

[23] Zacharin RF (1992). Free full-thickness vaginal epithelium graft in correction of recurrent genital prolapse. Aust N Z J Obstet Gynaecol..

[24] Rovner ES, Ginsberg DA (2001). Posterior vaginal wall prolapse: transvaginal repair of pelvic floor relaxation, rectocele, and perineal laxity. Tech Urol..

[25] Abramov Y (2005). Site-specific rectocele repair compared with standard posterior colporrhaphy. Obstet Gynecol..

